# Liposomal Ellagic Acid Alleviates Cyclophosphamide-Induced Toxicity and Eliminates the Systemic *Cryptococcus neoformans* Infection in Leukopenic Mice

**DOI:** 10.3390/pharmaceutics13060882

**Published:** 2021-06-15

**Authors:** Masood Alam Khan, Arif Khan, Mohd Azam, Khaled S. Allemailem, Faris Alrumaihi, Ahmad Almatroudi, Fahad A. Alhumaydhi, Faizul Azam, Shaheer Hasan Khan, Syeda Fauzia Farheen Zofair, Sumbul Ahmad, Hina Younus

**Affiliations:** 1 Department of Basic Health Sciences, College of Applied Medical Sciences, Qassim University, Buraydah 51452, Saudi Arabia; a_khan@qu.edu.sa (M.A.K.); 4140@qu.edu.sa (A.K.); 2 Department of Medical Laboratories, College of Applied Medical Sciences, Qassim University, Buraydah 51452, Saudi Arabia; mdazam25@gmail.com (M.A.); K.allemailem@qu.edu.sa (K.S.A.); f_alrumaihi@qu.edu.sa (F.A.); aamtrody@qu.edu.sa (A.A.); f.alhumaydhi@qu.edu.sa (F.A.A.); 3 Department of Pharmaceutical Chemistry & Pharmacognosy, Unaizah College of Pharmacy, Qassim University, Buraydah 51452, Saudi Arabia; faizulazam@gmail.com; 4 Interdisciplinary Biotechnology Unit, Faculty of Life Sciences, Aligarh Muslim University, Aligarh 202002, India; hasankhan14678@gmail.com (S.H.K.); fauziafarheen.786@gmail.com (S.F.F.Z.); ahmad.sumbul@yahoo.co.in (S.A.)

**Keywords:** *C. neoformans*, fluconazole, ellagic acid, liposomes, leukopenia

## Abstract

*Cryptococcus neoformans* infections rose sharply due to rapid increase in the numbers of immunocompromised individuals in recent years. Treatment of Cryptococcosis in immunocompromised persons is largely very challenging and hopeless. Hence, this study aimed to determine the activity of ellagic acid (EA) in the treatment of *C. neoformans* in cyclophosphamide injected leukopenic mice. A liposomal formulation of ellagic acid (Lip-EA) was prepared and characterized, and its antifungal activity was assessed in comparison to fluconazole (FLZ). The efficacy of the drug treatment was tested by assessing survival rate, fungal burden, and histological analysis in lung tissues. The safety of the drug formulations was tested by investigating hepatic, renal function, and antioxidant levels. The results of the present work demonstrated that Lip-EA, not FLZ, effectively eliminated *C. neoformans* infection in the leukopenic mice. Mice treated with Lip-EA (40 mg/kg) showed 70% survival rate and highly reduced fungal burden in their lung tissues, whereas the mice treated with FLZ (40 mg/kg) had 20% survival rate and greater fungal load in their lungs. Noteworthy, Lip-EA treatment alleviated cyclophosphamide-induced toxicity and restored hepatic and renal function parameters. Moreover, Lip-EA treatment restored the levels of superoxide dismutase and reduced glutathione and catalase in the lung tissues. The effect of FLZ or EA or Lip-EA against *C. neoformans* infection was assessed by the histological analysis of lung tissues. Lip-EA effectively reduced influx of inflammatory cells, thickening of alveolar walls, congestion, and hemorrhage. The findings of the present study suggest that Lip-EA may prove to be a promising therapeutic formulation against *C. neoformans* in immunocompromised persons.

## 1. Introduction

Immunocompromised persons, including AIDS patients, cancer patients, and persons undergoing organ transplantation are easy targets for opportunistic fungal pathogens [1]. A majority of fungal infections occur in immunosuppressed persons, but *C. neoformans* can cause infection in immunocompetent and immunocompromised individuals as well [2]. *C. neoformans* adopts the unique virulence factors and strategies to evade the host’s immune mechanisms [3]. The lungs are common sites of *C. neoformans* infection, but *C. neoformans* infection of the brain causes more than 60% mortality rate in the infected persons [4]. Innate immune responses, particularly neutrophils and macrophages, protect the host against *C. neoformans* through the generation of reactive oxygen species and reactive nitrogen species [5,6,7]. Various antifungal agents such as amphotericin B, itraconazole, fluconazole, and flucytosine can be used in the treatment of *C. neoformans* infection. Although the use of azole antifungals is considered safe, the emergence of azole-resistant *C. neoformans* rendered them ineffective. Amphotericin B is considered a “gold standard” drug to treat fungal infectious diseases. However, it exerts serious renal and hepatic toxicity in the treated subjects [8].

*Cryptococcus neoformans* contains several virulence factors, including melanin, capsule components, glycosyltransferases, cryptococcal xylosyltransferase 1, mannosyltransferases, and laccase [9]. Laccase is an important virulence factor that plays a role in melanin formation and functions to protect *C. neoformans* against the onslaught of the host immune response [10]. The melanized *C. neoformans* showed less susceptibility to amphotericin B and caspofungin [11]. Laccase-mediated immune modulation was suggested to play a critical role in the dissemination of *C. neoformans* infection in the brain tissues, as the deletion of Lac 1 gene prevented the brain dissemination of *C. neoformans* [12].

In recent years, the plant-derived compounds were shown to possess potential therapeutic benefits in the treatment of various infectious diseases [13,14]. Nevertheless, a major limitation with the use of phytochemicals is their low bioavailability that hinders their therapeutic use in the clinical setting. Ellagic acid (EA), a polyphenolic compound present in various fruits and vegetables, exhibits anti-oxidant, anti-inflammatory, and other therapeutic benefits [15]. Moreover, EA was shown to be effective against various pathogens, including bacteria, fungi, and parasites [16,17,18,19,20]. EA showed a synergistic effect with chloroquine in the treatment of malaria [19]. Antiviral drugs oseltamivir and isoprinosine in combination with EA showed potent antiviral activity against H3N2 viral infection [21]. EA alleviated diabetes-associated complications due to its strong antioxidant property [22]. Due to an increase in the numbers of immunocompromised individuals, there is a continuous surge in the frequency and the occurrence of fungal infections in recent years [23]. Currently, there are limited options of antifungal agents that can be used to treat the invasive fungal infections. Therefore, we need to search for new antifungal chemotherapeutic agents that are effective and less toxic, particularly in immunocompromised individuals. Although EA showed promising activity in the treatment of various infections, its efficacy was found to be very limited due to its poor solubility in an aqueous medium. In order to increase its therapeutic efficacy, we prepared a formulation of liposomal EA (Lip-EA) and assessed its therapeutic activity against *C. neoformans*. The results of the current study demonstrated that, as compared to FLZ or free EA, Lip-EA alleviated cyclophosphamide-induced toxicity and eliminated the systemic cryptococcosis in the treated mice.

## 2. Materials and Methods

The 1,2-dipalmitoyl-Sn-glycero-3-phosphocholine (DPPC) and the cholesterol were purchased from the Avanti Polar Lipids (Alabaster, AL, USA). Sabouraud dextrose agar (SDA), peptone, and yeast extracts were purchased from the HiMedia Company (Mumbai, India). Cyclophosphamide, ellagic acid, fluconazole, and RPMI were purchased from Sigma-Aldrich (St. Louis, MO, USA). AST, ALT, BUN, creatinine, SOD, catalase, and GSH estimation kits were purchased from the Abcam (Cambridge, UK).

### 2.1. Cryptococcus Neoformans

The clinical isolate of *C. neoformans* was obtained from the microbiology laboratory of the King Fahad Specialist Hospital, Buraydah, Saudi Arabia. It was maintained on sabouraud dextrose agar (SDA) plates. *C. neoformans* was identified by the positive urease and laccase test.

### 2.2. Antifungal Susceptibility Testing

The minimum inhibitory concentration (MIC) of EA or FLZ was determined by the microdilution method as per the guidelines of CLSI document M27-A3 [24]. To determine their MICs against *C. neoformans*, EA and FLZ were tested over a concentration range of 0.20 to 200 μg/mL in a 96-well plate. *C. neoformans* suspension was prepared in RPMI-1640 medium and adjusted to an inoculum of 2 × 10^3^ cells/mL. The wells containing *C. neoformans* with various concentrations of EA or FLZ and proper control were incubated at 37 °C for 48 h. The MIC was considered the lowest concentration of EA or FLZ that caused 50% inhibition as compared to the control growth without drugs.

### 2.3. Analysis of C. neoformans Death by the Confocal Microscopy

*Cryptococcus neoformans* (1 × 10^6^ CFUs) was cultured in Tryptic soya broth (TSB) in the presence or the absence of FLZ (1, 2, 4, and 8 μg/mL) or EA (2, 4, 8, and 16 μg/mL) for 48 h. After gentle washing with PBS, the cells were stained with propidium iodide (PI) for 30 min. After washing, the cells were analyzed by the confocal microscopy using a 20× magnification objective as described earlier [25].

### 2.4. Time Kill Curve Analysis

Time kill assay was performed with 5 × 10^5^ CFUs/mL of *C. neoformans* with 4, 8, 16, and 32 µg/mL of FLZ or EA as described earlier [26]**.** *C. neoformans* was cultured in the TSB and incubated at 37 °C for 48 h. *C. neoformans* suspension (5 × 10^5^ CFUs) was transferred to the flasks containing 20 mL of the TSB and above mentioned amounts of FLZ or EA. Samples (100 µL) were aseptically taken in duplicates at different time intervals (0, 3, 6, 12, and 24 h) and centrifuged at 10,000 rpm for 15 min. In order to minimize any drug carryover effect, the pellet of *C. neoformans* was reconstituted with sterile PBS. The numbers of CFUs were quantified by plating the serial dilutions onto SDA.

### 2.5. Determination of the Effect of EA or FLZ on the Preformed Biofilm Formation in C. neoformans

The effect of EA or FLZ against the preformed biofilm by *C. neoformans* was analyzed as described earlier [26,27]. *C. neoformans* was cultured in the TSB to obtain approximately 1 × 10^6^ CFUs/mL. *C. neoformans* (100 µL) was inoculated into a 96-well plate and incubated at 37 °C for 48 h. Without disrupting the biofilm, the culture medium was replaced with a fresh TSB containing EA or FLZ (16, 32, and 64 µg/mL). The plates were incubated overnight at 37 °C. Subsequently, the plate was gently washed with sterile phosphate-buffered saline (PBS) to remove free floating *C. neoformans*. The wells containing the biofilms were stained with 0.1% crystal violet solution. The plates were washed, dried and 95% ethanol was added to each well to solubilize the film. The extent of the biofilm formation was measured at 595 nm

### 2.6. Preparation of Liposomal EA (Lip-EA)

DPPC and cholesterol (7:3 molar ratio) were dissolved in a mixture of chloroform and methanol as described earlier [28]. Lipids and EA were taken in a molar ratio of 10:1. The solvent containing Lipids and EA was evaporated to form the dried lipid film. The lipid film was hydrated with an appropriate amount of sterile PBS followed by a brief sonication. The suspension was sonicated to prepare nano-sized lipid particles containing EA. Free EA was separated from the liposomal formulation by centrifugation at 15,000 rpm for 30 min. The final concentrations of EA and lipids were adjusted to 4.5 mg and 70 mg per ml of liposomal formulation.

### 2.7. Characterization of Liposomes

Liposomes were passed 3× through a 100 nm size of the membrane using a miniextruder device from the Avanti polar lipids (Alabaster, AL, USA). The size and the shape of EA-liposomes were determined by the transmission electron microscopy (TEM). Samples were dropped on carbon-coated copper grid followed by staining and drying. The images were recorded on the high resolution TEM operating at 200 kV [8]. The size and the polydispersity index (PDI) of EA-loaded liposomes were also measured by the Malvern Nano Zeta Sizer (Malvern instruments, Southborough, MA, USA) using the dynamic light scattering (DLS) technique.

### 2.8. Determination of the Entrapment Efficiency of EA in Liposomes

The amount of free EA was separated from the Lip-EA by centrifugation at 15,000 rpm for 30 min. The amount of Lip-EA was measured by taking the absorbance at 340 nm. An aliquot (100 µL) of Lip-EA was disrupted in DMSO to quantify EA using the standard curve. The entrapment efficiency of EA was calculated by estimating the Lip-EA out of the total EA (Equation (1)).

Percent entrapment efficiency of EA = (Lip-EA/Total EA) × 100(1)

In order to assess the stability of EA liposomes, 1 mL of Lip-EA was taken in cellulose dialysis tubing in a beaker containing 25 mL of distilled water. One ml aliquot was taken from the beaker at 1, 2, 3, 6, 12, and 24 h post incubation. The absorbance of the aliquot was quantified at 330 nm to determine the leakage of EA.

### 2.9. Mice

Swiss male mice (age of 12–14 weeks and 24–28 g weight) were taken from the animal house facility of the King Saud University, Riyadh, Saudi Arabia [26]. The techniques used for bleeding, injection, and sacrifice of mice were approved by the Animal Ethics Committee of the College of Applied Medical Sciences, Qassim University, Saudi Arabia (Approval number: 5575-cams1-2019-2-2-I and approval date: 14 November 2019). Mice were kept in hygienic and pathogen-free conditions. Sick mice were instantly euthanized and killed by the cervical dislocation in order to reduce their suffering.

### 2.10. Cyclophosphamide-Induced Leukopenia

In order to induce leukopenia, each mouse was injected with cyclophosphamide at a dose of 200 mg/kg through the intraperitoneal route. On day 4 post-cyclophosphamide treatment, the blood was taken from the mice, and the total numbers of leukocytes, erythrocytes and platelets were counted using the hematology analyzer.

### 2.11. Infection of Leukopenic Mice with C. neoformans

Since mice showed leukopenia on day 4 post-cyclophosphmaide injection, mice were infected with 7 × 10^5^ CFUs of *C. neoformans* through the lateral tail vein on the same day [29].

### 2.12. FLZ Therapy of C. neoformans Infected Mice

After 24 h of *C. neoformans* infection, mice were treated with various doses of FLZ through the intraperitoneal route for a week. Mice were divided into four groups, and each group contained 10 mice (Table 1).

### 2.13. Assessment of Antifungal Activity of EA and Lip-EA

The efficacy of EA or Lip-EA (20 and 40 mg/kg) was assessed against *C*. *neoformans*. A single daily dose of EA or Lip-EA was administered in mice for a week. Mice were divided into six groups, and each group contained ten mice (Table 2)

### 2.14. Evaluation of the Severity of C. neoformans Infection

The antifungal efficacy of FLZ or EA or Lip-EA against *C. neoformans* was evaluated by the survival rate and the fungal load in the lung tissues [29]. Mice were observed for 40 days post infection. In order to determine the severity of the infection, three mice from each group were sacrificed on day 5 post-infection, and the lung tissues were excised. The lung tissues were homogenized in cold PBS, and the tissue homogenates were plated on SDA plates. The plates were incubated for 48 h at 37 °C. The CFUs were counted and multiplied by the dilution factor.

### 2.15. Evaluation of Toxicity

In order to determine the hepatic and renal toxicity, the blood as taken from three mice of each group to determine aspartate transaminase (AST), alanine transaminase (ALT), blood urea nitrogen (BUN), and creatinine [30]. Moreover, the status of the oxidative stress in the infected mice was analyzed by determining superoxide dismutase (SOD), catalase (Cat), and reduced glutathione (GSH) in the lung tissue homogenate.

### 2.16. Analysis of the Airway Inflammation by the Histological Studies

In order to perform the histological analysis, the lung tissues from normal, untreated-infected, FLZ treated, EA-treated, or Lip-treated mice were fixed using 10% buffered formalin solution, and the paraffin-embedded blocks were made. The sections of 5 μm thickness were sliced and stained with mucicarmine staining [30]. The slides were studied under the light microscope (Leica Inc., Buffalo Grove, IL, USA) at 400× magnification to observe the infection-induced changes in the lung tissues from untreated or treated mice.

### 2.17. Statistical Analyses

Survival data are shown by Kaplan–Meier curve and were analyzed by the Log-rank Chi square test. The fungal burden data were analyzed by one-way ANOVA followed by a Bonferroni post test using GraphPad Prism software, version 6.0 (La Jolla, CA, USA).

## 3. Results

### 3.1. In Vitro Activity of EA against C. neoformans

EA exhibited a significant activity against *C. neoformans,* as shown by the MIC results. The MIC of EA against the present *C. neoformans* isolate was found to be 16 µg/mL, whereas the MIC of FLZ was found to be 8 µg/mL.

### 3.2. The Confocal Microscopy Analysis Revealed an Antifungal Activity of EA against C. neoformans

**Figure 1:**
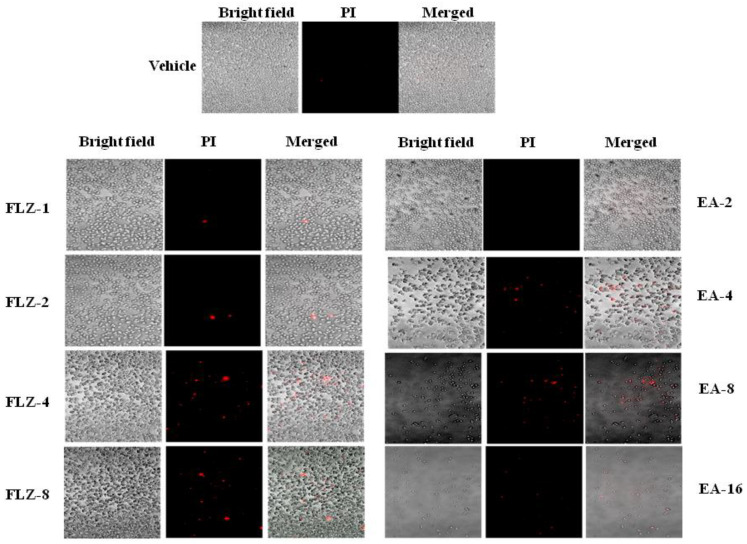
The effect of FLZ or EA on the cell viability of *C. neoformans* by the confocal microscopy using PI staining.

The antifungal effect of FLZ or EA against *C. neoformans* was measured by the confocal microscopy. FLZ showed a dose-dependent activity against *C. neoformans,* as shown by PI-positive cells (Figure 1). As compared to FLZ, EA was more effective against *C. neoformans* at the comparable MIC values. A continued reduction was observed in the viability of *C. neoformans* yeast cells treated with EA (Figure 1). However, no PI^+^ cells were detected in vehicle-treated *C. neoformans*.

### 3.3. EA Showed Activity against C. neoformans by Time Kill Studies

**Figure 2:**
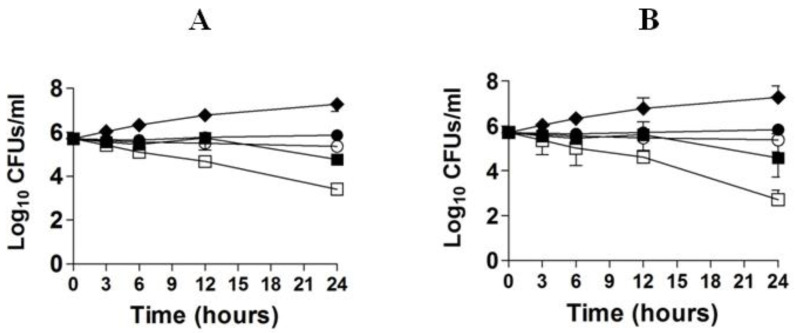
Time kill curves of FLZ (**A**) or EA (**B**) against *C. neoformans*. (**A**) Control (♦), FLZ-4 µg/mL (⚫), FLZ-8 µg/mL (◯), FLZ-16 µg/mL (■), FLZ-32 µg/mL (☐). (**B**) Control (♦), EA-4 µg/mL (⚫), EA-8 µg/mL (◯), EA-16 µg/mL (■), EA-32 µg/mL (☐). The data are represented as mean ± SD of three independent experiments.

The in vitro activity of FLZ or EA against *C. neoformans* was studied by time kill studies. The results of time kill study demonstrated that there was a time-dependent and a dose-dependent effect of FLZ or EA against *C. neoformans* (Figure 2A,B). FLZ at the concentrations of 16 and 32 µg/mL showed greater fungicidal activity against *C. neoformans* at the 24 h time point. FLZ at 32 µg/mL reduced ≥3 log10 CFUs/mL of *C. neoformans* as compared to the control group at the 24 h time point (Figure 2A). FLZ at the concentrations of 8 and 16 µg/mL killed 55.4% and 88.7% of the original inoculum of *C. neoformans*. However, EA showed greater fungicidal activity against *C. neoformans* at the comparable concentrations. EA at the concentration of 32 µg/mL reduced >4 log10 CFUs/mL of *C. neoformans* as compared to the CFUs in the control group at the 24 h time point, whereas EA at the concentration of 16 µg/mL killed 94.7% of original *C. neoformans* inoculum (Figure 2B).

### 3.4. EA Effectively Inhibited the Preformed Biofilm in C. neoformans

**Figure 3:**
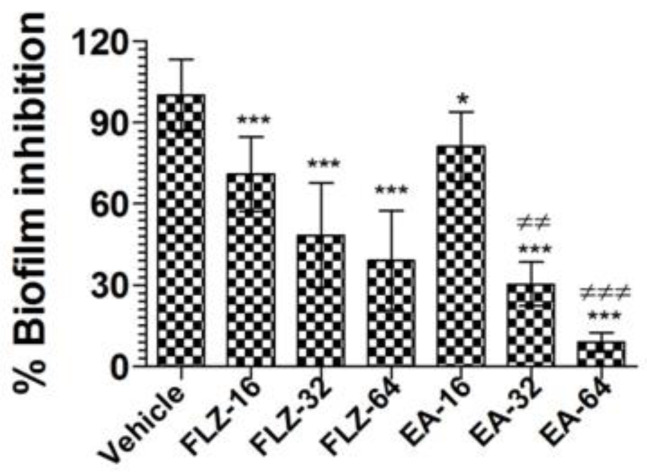
The activity of FLZ and EA against the preformed biofilm of *C. neoformans*. A *p* value < 0.05 was considered to be significant. * (*p* < 0.05), *** (*p* < 0.001) FLZ or EA treatment vs. vehicle treatment. ^≠≠^ *p* < 0.01 and ^≠≠≠^ *p* < 0.001, EA-treated groups vs. FLZ treated groups. The data are represented as mean ± SD of three independent experiments.

Treatment with EA or FLZ at higher concentration was effective in eradicating the preformed biofilm of *C. neoformans* (Figure 3). FLZ (32 and 64 µg/mL) caused 52% and 61% eradication of the preformed biofilm. Interestingly, EA at the same concentration eradicated 70% and 91% of the preformed biofilm, respectively (Figure 3) (*p* < 0.001). It suggested that EA was more effective to remove the preformed biofilm at the similar concentrations. Noteworthy, FLZ at a concentration of 64 µg/mL (eight-fold of the MIC) caused 61% eradication, whereas EA at the same concentration (four-fold of the MIC) eliminated 91% of the preformed biofilm (*p* < 0.001).

### 3.5. EA-Liposomes Showed Greater Stability in an Aqueous Medium

**Figure 4:**
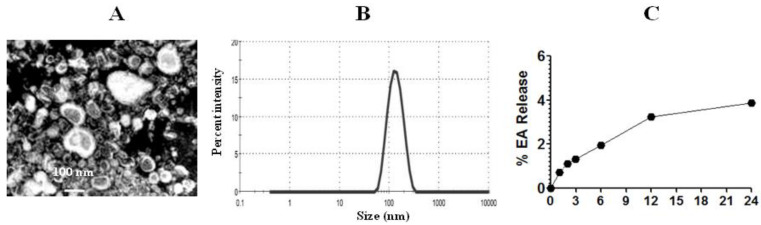
Size and stability of liposomes. (**A**) TEM image of EA liposomes, (**B**) size of EA liposomes, (**C**) stability of EA liposomes.

The diameter of EA liposomes was measured by TEM and DLS techniques (Figure 4A). PDI lies between zero and one, and a lower PDI value suggests more homogeneity and greater stability. The PDI value of EA-loaded liposomes was 0.375. The size of EA liposomes was found to be in the range of 50–200 nm (Figure 4B). The entrapment efficiency of EA in liposomes was found to be 62.4%. The stability of Lip-EA was determined in distilled water. As shown in Figure 4C, the releases of EA were found to be 3.24% and 3.88% at 12 h and 24 h time points, respectively. The small leakage of EA from the liposomal formulation suggests that EA is strongly associated with liposomes.

### 3.6. Cyclophosphamide Induces the Depletion of Leukocytes, Erythrocytes, and Platelets

**Figure 5:**
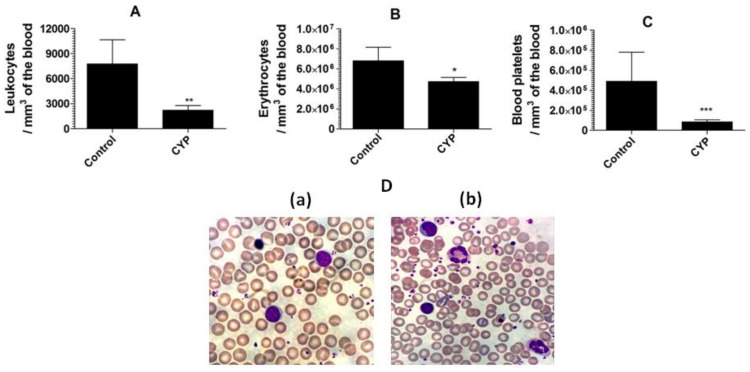
Cyclophosphamide administration decreases the numbers of the leukocytes, erythrocytes, and platelets in mice. (**A**) Leukocytes, (**B**) erythrocytes, (**C**) blood platelets. Data are expressed as mean ± SD. A *p* value < 0.05 was considered to be significant. * (*p* < 0.05), ** (*p* < 0.01), *** (*p* < 0.001). (**D**(**a**)) Blood picture from control mice, (**D**(**b**)) blood picture from cyclophosphamide injected mice.

Cyclophosphamide caused a substantial reduction in the leukocyte numbers to 2221 ± 558 as compared to the leukocyte numbers of 7751 ± 678 per mm^3^ in the blood from normal mice (Figure 5A) (*p* = 0.0033). Moreover, cyclophosphamide administration also reduced the numbers of erythrocyte and blood platelet (Figure 5B,C). Mice in the control group showed erythrocyte numbers of 6,793,000 ± 314,200/mm^3^ of the blood, whereas cyclophosphamide injected mice had erythrocyte numbers of 4,733,000 ± 421,000 (*p* = 0.0086). Noteworthy, the blood platelet count was extremely reduced in cyclophosphamide injected mice (Figure 5C). The normal mice had the blood platelet numbers of 489,900 ± 67,370, whereas cyclophosphamide injected mice had 85,790 ± 20,120 (*p* = 0.0045). The structural changes in the leukocytes were analyzed by studying the blood picture developed from normal control and cyclophosphamide injected mice. The leukocytes from the blood of the normal control mice demonstrated compactness and integrity in their structure (Figure 5D(a)), whereas, the leukocytes from cyclophosphamide injected mice showed degranulation, vacuolation, and disintegration of nuclei. Moreover, a greater platelet aggregation was observed in the blood picture from cyclophosphamide injected mice (Figure 5D(b)).

### 3.7. Treatment with Lip-EA, but Not with FLZ, Was Highly Effective against C. neoformans in Leukopenic Mice

**Figure 6:**
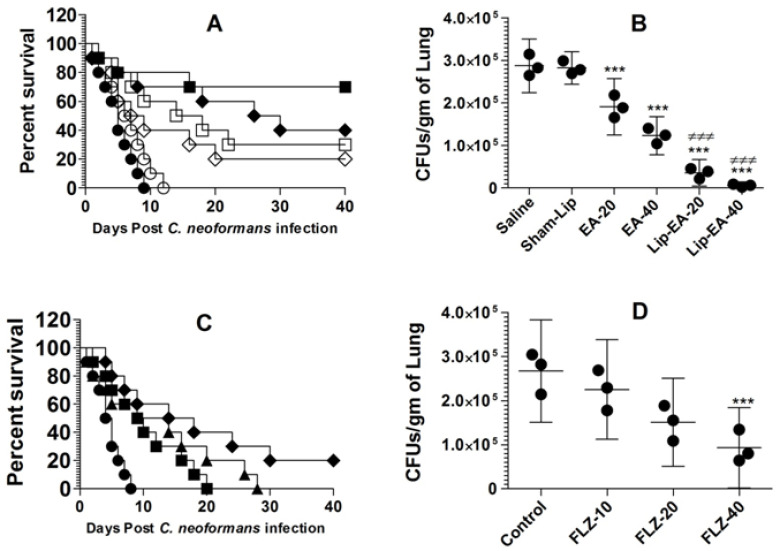
Treatment with Lip-EA, not FLZ, effectively eliminated cryptococcosis in leukopenic mice. (**A**) Mice were observed 40 days after treatment with EA or Lip-EA as described in the Methodology section. Saline (⚫), sham-lip (◯), EA-20 mg/kg (◇), EA-40 mg/kg (☐), Lip-EA-20 mg/kg (♦), Lip-EA-20 mg/kg (■). Saline vs. EA-20 mg/kg (*p* = 0.0370), saline vs. EA-40 mg/kg (*p* = 0.0029), sham-lip vs. Lip-EA-20 mg/kg (*p* = 0.0011), sham-lip vs. Lip-EA-40 mg/kg (*p* = 0.0005). (**B**) On day 5, three mice from each group were sacrificed, and their lungs were homogenized in order to determine the fungal load. Data are expressed as mean ± SD. A *p* value < 0.05 was considered to be significant. *** (*p* < 0.001) Treatment group vs. saline or sham-lip, ^≠≠≠^ (*p* < 0.001) Lip-EA vs. EA. (**C**) Mice were observed 40 days after treatment with FLZ as described in the Methodology section. Saline (⚫), FLZ-10 mg/kg (■), FLZ-20 mg/kg (▲), FLZ-40 mg/kg (♦). Saline vs. FLZ-10 mg/kg (*p* = 0.0193), saline vs. FLZ-20 mg/kg (*p* = 0.0166), saline vs. FLZ-40 mg/kg (*p* = 0.0021). (**D**) On day 5, three mice from each group were sacrificed, and their lungs were homogenized in order to determine the fungal load. Data are expressed as mean ± SD. *** (*p* < 0.001) FLZ-40 vs. control.

Because of its poor solubility in an aqueous medium, EA shows low bioavailability in animal models. In order to increase its bioavailability in an in vivo system, we used a liposomal formulation of EA in the treatment of murine cryptococcosis. The activity of EA or Lip-EA or FLZ was assessed against *C. neoformans* in leukopenic mice. *C. neoformans* infected mice treated with EA at the doses of 20 and 40 mg/kg had 20% and 30% survival rate on day 40 post-infection, whereas the mice in the saline-treated group died within 10 days post-infection (*p* = 0.0370 and *p* = 0.003, respectively) (Figure 6A). Lip-EA showed greater activity against *C. neoformans*. *C. neoformans* infected mice had 40% and 70% survival rate in the groups treated with 20 and 40 mg/kg of Lip-EA, whereas all mice treated with sham-lip died within 12 days post-infection (*p* < 0.01 and *p* < 0.001, respectively) (Figure 6A). The severity of cryptococosis was assessed by determining the fungal load in the lung tissue. The culture of the lung tissue homogenate revealed the fungal load of 287,125 ± 25,369 CFUs in the saline-treated mice (Figure 6B). The mice treated with EA (20 and 40 mg/kg) had the fungal burden of 190,935 ± 26,661 and 122,895 ± 18,019 CFUs per gram of the tissue (*p* < 0.01 and *p* < 0.001, respectively). However, treatment with Lip-EA (20 and 40 mg/kg) was highly effective to reduce CFUs to 85,254 ± 19,983 and 19,993 ± 10,268 as compared to 281,983 ± 15,435 CFUs in sham-lip treated mice (*p* < 0.001). Moreover, the efficacy of Lip-EA (40 mg/kg) was significantly higher in comparison to EA treatment (*p* < 0.001).

The activity of FLZ at the doses of 10, 20, and 40 mg/kg was assessed against *C. neoformans* infection in immunocompromised mice. *C. neoformans* infected leukopenic mice did not respond well to FLZ therapy, and all infected mice treated with FLZ at the doses of 10 and 20 mg/kg died within 40 days post-infection (Figure 6C). The median survival time (MST) of mice in the saline-treated group was 4.5 days, whereas the MST of mice in the group treated with FLZ (10 mg/kg) was found to be 9.5 days (Figure 6C). The MSTs of mice in the groups treated with FLZ at the doses of 20 and 40 mg/kg were found to be 11.5 and 16 days (*p* = 0.0166 and *p* = 0.0021, respectively). Treatment with FLZ at a dose of 40 mg/kg showed 20% survival rate at day 40 post-infection (Figure 6C). The culture of the lung tissue homogenate revealed the fungal load of 267,125 ± 46,862 CFUs in the saline-treated mice (Figure 6D), whereas the mice treated with FLZ (10 and 20 mg/kg) had the fungal burdens of 225,316 ± 45,541 and 150,935 ± 40,188 CFUs, which were found to be insignificant as compared to CFUs in the lung tissues of the saline-treated mice (*p* > 0.05). However, the treatment with FLZ at a dose of 40 mg/kg significantly reduced CFUs to 92,895 ± 36,686 (*p* < 0.01).

### 3.8. Treatment with Lip-EA Alleviated, Whereas FLZ Exacerbated, Hepatic and Renal Toxicity

**Figure 7:**
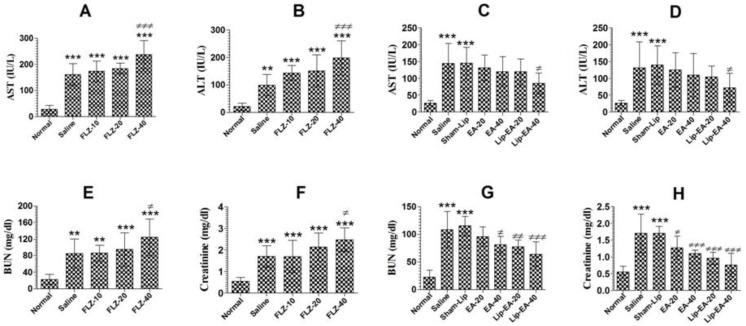
EA or Lip-EA, not FLZ, alleviated cyclophosphamide-induced toxicity in *C. neoformans* infected mice. (**A**,**B**) FLZ treatment increased the levels of AST and ALT in treated mice. *** (*p* < 0.001) Normal mice vs. saline or FLZ treated mice, ^≠≠≠^ *p* < 0.001 saline treatment vs. FLZ-40 treatment. (**C**,**D**) Treatment with EA or Lip-EA normalizes the levels of AST and ALT. *** (*p* < 0.001) Normal mice vs. saline or sham-lip treated mice, ^≠^ *p* < 0.05 sham-lip treated mice vs. Lip-EA-40 treated mice. (**E**,**F**) FLZ treatment increased the levels of BUN and creatinine in treated mice. ** (*p* < 0.01) Normal mice vs. saline or FLZ-10 treated mice, *** (*p* < 0.001) normal mice vs. FLZ-20 or FLZ-40 treated mice, *** (*p* < 0.001) normal mice vs. saline or FLZ treated mice, ^≠^ *p* < 0.05 saline treatment vs. FLZ-40 treatment. (**G**,**H**) Treatment with EA or Lip-EA normalizes the levels of BUN and serum creatinine. For BUN: *** (*p* < 0.001) normal mice vs. saline or sham-lip treated mice, ≠ *p* < 0.05 saline-treated mice vs. EA-40 treated mice, ^≠≠^ *p* < 0.01 sham-lip treated mice vs. Lip-EA-20 treated mice, ^≠≠≠^ *p* < 0.001 sham-lip treated mice vs. Lip-EA-40 treated mice; for creatinine: *** (*p* < 0.001) normal mice vs. saline or sham-lip treated mice, ^≠^ *p* < 0.05 saline-treated mice vs. EA-20 treated mice, ^≠≠≠^ *p* < 0.001 saline-treated mice vs. EA-40 treated mice, ^≠≠≠^ *p* < 0.001 sham-lip-treated mice vs. Lip-EA-20 or Lip-EA-40 treated mice.

Cyclophosphamide administration induces leukopenia and toxicity in mice. FLZ treatment elevated the levels of AST and ALT in *C. neoformans* infected leukopenic mice (Figure 7A). The level of AST increased from 27.33 in the normal mice to 160.7 IU/L in the saline treated mice (*p* < 0.001). Treatment with FLZ at a dose of 40 mg/kg further increased the AST level from 160.7 to 237 IU/L (Figure 7A) (*p* < 0.001). As with AST, the level of the ALT was also increased from 21.3 to 99.3 IU/L in cyclophosphamide injected mice (Figure 7B). Treatment with FLZ (40 mg/kg) further increased the ALT level from 99.3 to 198.6 IU/L (Figure 7B).

Treatment with EA (20 and 40 mg/kg) alleviated the hepatic toxicity by reducing the AST levels to 131.7 ± 15.18 and 119.7 ± 18 IU/L (Figure 7C) (*p* > 0.05). However, Lip-EA treatment at a dose of 40 mg/kg was more effective and reduced AST to 85.33 ± 12.22 (*p* < 0.05). Moreover, the ALT level was increased from 26.67 ± 3 in normal mice to 131.7 ± 30.9 IU/L in saline-treated mice (Figure 7D). Treatment with EA at the doses of 20 and 40 mg/kg reduced ALT levels to 125 ± 20 IU/L and 109 ± 25 IU/L (*p* > 0.05) (Figure 7D). Noteworthy, Lip-EA at a dose of 40 mg/kg reduced ALT to 72 ± 17 IU/L as compared to that of 139.7 ± 23 IUL/L in sham-lip treated mice (*p* < 0.05).

Treatment with FLZ induced renal toxicity, as demonstrated by the greater levels of BUN and creatinine (Figure 7E,F). The level of BUN increased from 21.3 mg/dL in normal mice to 85.3 mg/dL in *C. neoformans* infected saline-treated mice (Figure 7E) (*p* < 0.01). Treatment with FLZ (10, 20 and 40 mg/kg) further increased the BUN level as compared to normal mice (*p* < 0.001). Furthermore, FLZ at a dose of 40 mg/kg significantly increased the BUN level to 125 mg/dL (Figure 7E) (*p* < 0.05). As with the BUN, the creatinine level was also significantly increased in saline-treated and FLZ treated mice (Figure 7F) (*p* < 0.001). There was a significant elevation in creatinine level to 2.47 mg/dL in mice treated with FLZ (40 mg/kg) as compared to that of 1.7 mg/dL in the saline treated mice (Figure 7F) (*p* < 0.05).

Treatment with EA or Lip-EA improved the parameters of the renal toxicity, as shown by a decline in the levels of BUN and creatinine (Figure 7G,H). The level of BUN increased from 23 ± 5 mg/dL in the normal mice to 109 ± 13 mg/dL in *C. neoformans* infected saline treated mice (Figure 7G) (*p* < 0.001). Treatment of mice with EA (20 and 40 mg/kg) decreased the BUN levels to 96 ± 7 (*p* > 0.05) and 81 ± 6 mg/dL (*p* < 0.05), respectively. Furthermore, Lip-EA treatment at the doses of 20 and 40 mg/kg significantly decreased the BUN levels to 78 ± 5 and 64 ± 9 mg/dL as compared to the BUN level of 115 ± 7 mg/dL in sham-lip treated mice (Figure 7G) (*p* < 0.01 and *p* < 0.001, respectively). Similar to BUN, the creatinine level was also significantly alleviated in the mice treated with EA or Lip-EA at the doses of 20 and 40 mg/kg (Figure 7H). The administration of EA at the doses of 20 and 40 mg/kg decreased creatinine levels from 1.703 ± 0.23 mg/dL to 1.27 ± 0.14 and 1.1 ± 0.04 mg/dL (*p* < 0.05 and *p* < 0.001). Interestingly, Lip-EA treatment resulted in greater alleviation in creatinine levels, and the mice treated with Lip-EA (20 and 40 mg/dL) had 0.96 ± 0.09 and 0.76 ± 0.07 mg/dL, respectively (*p* < 0.001).

### 3.9. Treatment with EA or Lip-EA, Not FLZ, Relieves the State of Oxidative Stress in the Lung Tissues

**Figure 8:**
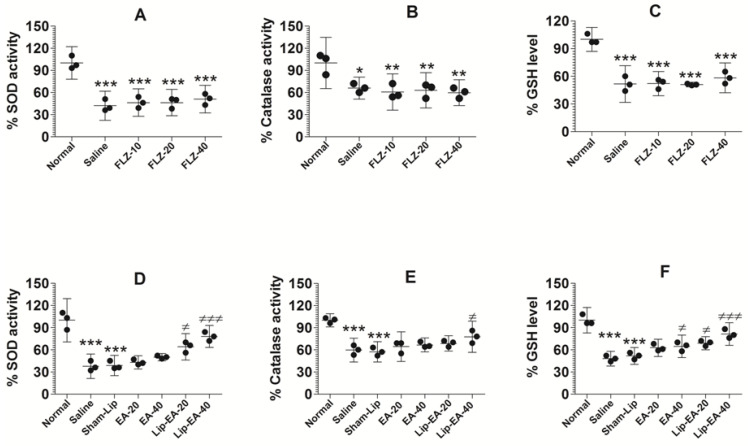
Treatment with EA or Lip-EA, not FLZ, relieved the state of oxidative stress in the lung tissues. (**A**) FLZ treatment decreased SOD activity. *** (*p* < 0.001) Normal mice vs. saline or FLZ treated mice. (**B**) FLZ treatment decreased catalase activity. * (*p* < 0.05) Normal mice vs. saline treated mice, ** (*p* < 0.01) normal mice vs. FLZ treated mice. (**C**) FLZ treatment decreased GSH activity. *** (*p* < 0.001) Normal mice vs. saline or FLZ treated mice. (**D**) Lip-EA treatment significantly restored SOD activity. *** (*p* < 0.001) Normal mice vs. saline or sham-lip treated mice, ^≠^ *p* < 0.05 sham-lip treated mice vs. Lip-EA-20 treated mice, ^≠≠≠^ *p* < 0.001 sham-lip treated mice vs. Lip-EA-40 treated mice. (**E**) Lip-EA treatment significantly restored catalase activity. *** (*p* < 0.001) Normal mice vs. saline or sham-lip treated mice, ^≠^ *p* < 0.05 sham-lip treated mice vs. Lip-EA-40 treated mice. (**F**) Lip-EA treatment significantly restored GSH activity. *** (*p* < 0.001) Normal mice vs. saline or sham-lip treated mice, ^≠^ *p* < 0.05 saline treated mice vs. EA-40 treated mice, ^≠^ *p* < 0.05 sham-lip treated mice vs. Lip-EA-20 treated mice, ^≠≠≠^ *p* < 0.001 sham-lip-treated mice vs. Lip-EA-40 treated mice.

Cyclophosphamide injected leukopenic mice had lower levels of antioxidant enzymes such as SOD, GSH, and catalase (Figure 8). The activity of SOD was found to be 37.7% in the lung tissues of saline-treated mice as compared to normal control mice (Figure 8A) (*p* < 0.001). The results showed that EA at the doses of 20 and 40 mg/kg did not alter the activity of SOD in *C. neoformans* infected mice. Conversely, the treatment with Lip-EA (20 and 40 mg/kg) significantly increased the activity of SOD in the lung tissue of mice as compared to that of the sham-lip treated mice (*p* < 0.05, *p* < 0.001). As with the activity of SOD, catalase activity was reduced to 60% in the lungs of saline treated mice as compared to the normal mice (Figure 8B) (*p* < 0.001). EA treatment could not significantly restore catalase activity as compared to that in saline treated mice (Figure 8B), whereas the mice treated with Lip-EA at a dose of 40 mg/kg had 78% catalase activity in lung tissues (*p* < 0.05). As with SOD and catalase activity, the level of GSH in the lung of cyclophosphamide injected mice was reduced to 48% of the GSH level in the lungs of normal control mice (Figure 8C) (*p* < 0.001). Treatment with EA at a dose 40 mg/kg elevated the GSH level from 48% to 66% (*p* < 0.05). Importantly, Lip-EA treatment (20 and 40 mg/kg) elevated GSH levels to 70% and 81%, which were significantly higher than the GSH level in the lung tissues of sham-lip treated mice (*p* < 0.05 and *p* < 0.001, respectively).

Treatment with FLZ with the doses of 10, 20, and 40 mg/kg did not alter the activity of SOD in *C. neoformans* infected leukopenic mice (Figure 8D). The activity of catalase was reduced in infected mice as compared to the normal mice (Figure 8E) (*p* < 0.05). FLZ treatment resulted in further reduction of catalase activity in the lung tissue homogenates as compared to catalase activity in saline-treated mice (Figure 8E). Similarly, the level of GSH was significantly reduced in cyclophosphamide injected mice as compared to normal mice (Figure 8F) (*p* < 0.001). However, FLZ treatment did not change the level of GSH in *C. neoformans* infected leukopenic mice (Figure 8F).

### 3.10. Treatment with Lip-EA Alleviated the Pathological Changes in the Lung Tissues of C. neoformans Infected Mice

**Figure 9:**
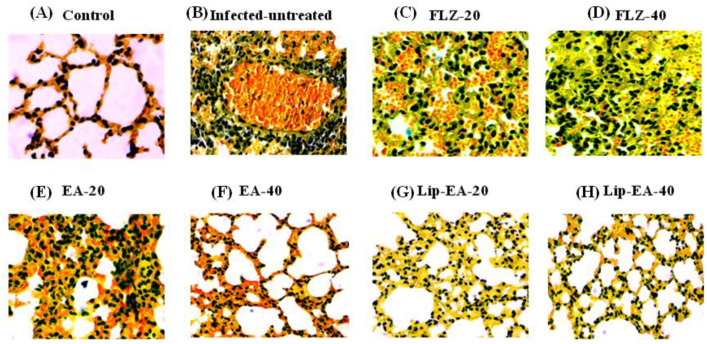
Treatment with Lip-EA alleviated the pathological changes in the lung tissues of *C. neoformans* infected leukopenic mice. Histological analysis of the lung tissues from (**A**) normal control, (**B**) untreated infected, (**C**) FLZ-20, (**D**) FLZ-40, (**E**) EA-20, (**F**) EA-40, (**G**) Lip-EA-20, (**H**) Lip-EA-40.

The findings of the histological analysis showed the pathological changes in the lung tissues of *C. neoformans* infected mice as compared to the lung tissues from the normal mice (Figure 9). There was highly increased infiltration of inflammatory cells, hemorrhage, and airway wall thickening in the lung tissues from the untreated *C. neoformans* infected mice (Figure 9). Treatment with FLZ (20 and 40 mg/kg) did not remarkably reduce the infiltration of inflammatory cells or the thickening of the airway (Figure 9). However, the treatment with Lip-EA at the doses of 20 and 40 mg/kg reduced the influx of inflammatory cells, congestion, and mucus secretion in the lung tissues of *C. neoformans* infected mice (Figure 9).

## 4. Discussion

In recent decades, *C. neoformans* infections increased dramatically due to an upsurge in the numbers of individuals with impaired immunity. Moreover, *C. neoformans* can also survive intracellularly and escape the onslaught of the host’s immune system [31]. Treatment of *C. neoformans* infected persons is very challenging due to drug-induced toxicity and poor immune response. Most of the antifungal drugs, including azoles and polyenes, induce renal toxicity in the treated patients. In order to find safe, effective, new therapeutic antifungal agents, we investigated the in vitro activity of EA against *C. neoformans*. In order to increase the therapeutic activity of EA, Lip-EA was prepared, and its activity was investigated against *C. neoformans* in leukopenic mice. The findings of the present study demonstrated that treatment with Lip-EA was more effective and safe as compared to FLZ in the treatment of the systemic cryptococcosis in immunocompromised mice.

Earlier studies demonstrated that EA and its complex with hydroxypropyl- β-cyclodextrin demonstrated its antifungal activity against *C. albicans* in Drosophila [18]. However, no study was performed to assess the antifungal activity of EA or Lip-EA in immunocompromised subjects. In the present study, we determined the activity of EA against *C. neoformans* and compared it with FLZ. Interestingly, EA exhibited an antifungal activity against *C. neoformans,* as demonstrated by the dilution method, the confocal microscopy analysis, and the time kill assay. The confocal microscopy analysis revealed that EA was more effective against *C. neoformans* as compared to FLZ at the comparable MIC values. *C. neoformans* treated with EA showed a higher ratio of PI+ to live cells as compared to FLZ treated *C. neoformans*. The findings of the confocal analysis were also supported by the time kill assay. The results of the time kill assay demonstrated that EA at a dose of 32 µg/mL killed more than 99% of *C. neoformans*. The formation of biofilm is considered an important strategy of *C. neoformans* to fight against the adverse conditions. The biofilm exhibited the resistance to antimicrobial agents and host immune defense [32]. EA and its derivatives alone or in combination with standard antibiotics demonstrated its activity against the biofilm formation by pathogens [33,34,35]. The activity of EA was found to be greater as compared to FLZ against the preformed biofilm by *C. neoformans*. The lower activity of FLZ against the preformed biofilm may be due to the fungistatic nature of the drug.

The lower solubility and bioavailability of EA are major limitations for its use in the treatment of various diseases. In order to increase its in vivo activity, nanoparticle-based formulations of EA were prepared and investigated in various disease models [36,37,38]. The encapsulation of EA in liposomes protects the drug and increases its hepatoprotective activity against cyclophosphamide -induced activity [37]. In the present study, we prepared Lip-EA and tested its activity against *C. neoformans* in leukopenic mice. Since *C. neoformans* infections are commonly found in immunocompromised subjects, we induced cyclophosphamide-induced immune suppression in mice. Cyclophosphamide injected mice showed severe leukopenia, as revealed by significantly lower numbers of leukocytes in their systemic circulation. Treatment of the opportunistic infections in immunocompromised subjects is very challenging, as they fail to respond to antibiotics [39]. Thus, it should be considered an important priority to find drug formulations that can effectively cure the opportunistic fungal infections in immunocompromised persons. Here, we investigated the activity of EA or Lip-EA against the systemic infection of *C. neoformans* in leukopenic mice. The findings showed that Lip-EA was very effective against *C. neoformans* infection as shown by the greater survival and the reduced fungal load in the lung tissues. The enhanced activity of Lip-EA can be attributed to higher solubility and bioavailability of EA in the liposomal form. As compared to FLZ, Lip-EA was found to be well tolerated in immunocompromised mice, as the administration of EA or Lip-EA alleviated the toxicity of cyclophosphamide. It was supported by an earlier study that showed the protective effect of EA against cyclophosphamide-induced renal toxicity [37,40]. The administration of FLZ, particularly at a dose of 40 mg/kg, exacerbated cyclophosphamide-induced toxicity that may be a possible reason for its reduced effectiveness to eliminate *C. neoformans* infection. Moreover, FLZ treatment elevated the levels of hepatic and renal toxicity parameters. Alternatively, EA or Lip-EA treatment alleviated the toxic effects of cyclophosphamide on liver and kidney functioning. In a recent report, Aslan et al. reported an anti-oxidant effect of EA against carbon tetrachloride-induced pulmonary toxicity [41]. Interestingly, the results of the current study showed that treatment with EA or Lip-EA, not with FLZ, increased the levels of SOD, Cat, and GSH that may protect against cyclophosphamide-generated free radical-induced toxicity.

We earlier showed that the influx of inflammatory cells contributes to congestion and inflammation of the lung tissues of OVA-induced allergic asthmatic mice [42]. The findings of the present study demonstrated that treatment with Lip-EA was very effective in reducing the numbers of inflammatory cells and the congestion in the lung tissues of *C. neoformans* infected mice. Alves et al. demonstrated that EA administration alleviated OVA-induced allergic airway inflammation in mice [43]. It suggests that EA or Lip-EA can be used as potential therapeutic agents in the treatment of lung inflammation-associated diseases.

In conclusion, EA showed activity against *C. neoformans* both in vitro and in a mouse model. Moreover, the administration of Lip-EA showed greater activity in alleviating cyclophosphamide-induced toxicity and in eliminating *C. neoformans* infection in a mouse model. The greater activity of Lip-EA was substantiated by increased survival rate and reduced fungal load. However, the treatment with FLZ aggravated cyclophosphamide-induced toxicity and therefore was ineffective to cure the systemic *C. neoformans* infection in the leukopenic mice. Collectively, these findings indicated that Lip-EA may prove to be an effective therapeutic formulation in the treatment of *C. neoformans* infection, particularly in immunocompromised subjects.

## Figures and Tables

**Table 1:** Distribution of mice in treatment groups.

S. No.	Treatment Group	No. of Mice
1	Normal saline	10
2	FLZ-10 mg/kg	10
3	FLZ-20 mg/kg	10
4	FLZ-40 mg/kg	10

**Table 2:** Distribution of mice in treatment groups.

S. No.	Treatment Group	No. of Mice
1	Normal saline	10
2	Sham liposomes	10
3	EA-20 mg/kg	10
4	EA-40 mg/kg	10
5	Lip-EA-20 mg/kg	10
6	Lip-EA-40 mg/kg	10

## Data Availability

All relevant data have been provided within the manuscript. There are no supporting files and no data was held.
